# Evaluation of the anti-tumor effects of lactate dehydrogenase inhibitor galloflavin in endometrial cancer cells

**DOI:** 10.1186/s13045-014-0097-x

**Published:** 2015-01-29

**Authors:** Xiaoyun Han, Xiugui Sheng, Hannah M Jones, Amanda L Jackson, Joshua Kilgore, Jessica E Stine, Monica N Schointuch, Chunxiao Zhou, Victoria L Bae-Jump

**Affiliations:** Department of Gynecologic Oncology, ShanDong Cancer Hospital & Cancer Institute, Jinan, 250117 P.R China; Division of Gynecologic Oncology, University of North Carolina at Chapel Hill, CB# 7572, Physicians Office Building Rm# B105, Chapel Hill, NC 27599 USA; Lineberger Comprehensive Cancer Center, University of North Carolina at Chapel Hill, Chapel Hill, NC USA

**Keywords:** Endometrial Cancer, LDH, Galloflavin, Glycolysis

## Abstract

High rates of aerobic glycolysis represent a key mechanism by which endometrial cancer cells consume glucose as its primary energy source. The up-regulated glycolytic pathway is a common therapeutic target whose inhibition has implications for anti-tumor activity in cancer cells. The present study was aimed at evaluating the potential of a novel lactate dehydrogenase (LDH) inhibitor, Galloflavin, as a therapeutic agent for endometrial cancer. Our results revealed that Galloflavin effectively inhibited cell growth in endometrial cancer cell lines and primary cultures of human endometrial cancer through its involvement in multiple signaling pathways that regulate metabolism, cell cycle, apoptosis, cell stress and metastasis.

**Electronic supplementary material:**

The online version of this article (doi:10.1186/s13045-014-0097-x) contains supplementary material, which is available to authorized users.

## To the Editor

**Figure 1:**
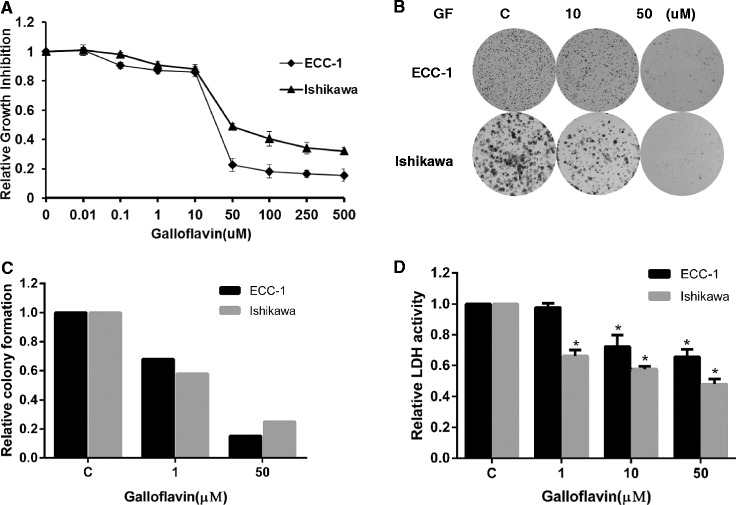
**Galloflavin inhibited cell proliferation in the ECC-1 and Ishikawa cells.** The ECC-1 and Ishikawa cell lines were cultured in 96-well plates for 24 hours and treated with GF at the indicated doses for 72 hours. Cell proliferation was assessed with MTT assay **(A)**. The cells were cultured in 6 well plates for 24 hours, treated with GF for 48 hours and then cultured for an additional 10 days. Colony formation assay was assessed **(B, C)**. The effect of GF on LDHA activity level was examined using a LDHA activity assay kit **(D)**. GF decreased LDHA activity in the endometrial cancer cells after 16 hours of treatment. (* < 0.05).

Galloflavin (GF), which is synthesized from gallic acid, is a new lactate dehydrogenase inhibitor that inhibits both the A and B isoforms of LDH [1]. By serving as a competitive inhibitor with NADH for LDH, GF has been shown to disrupt aerobic glycolysis and decrease cell viability effectively across many cancer cell types, including breast, colon and liver cancers as well as Burkitt lymphoma [2-4]. To investigate the effect of GF on endometrial cancer cell growth, we utilized the Ishikawa and ECC-1 cells exhibiting high rates of glycolysis to identify the potential of GF. GF significantly reduced LDHA activity, inhibited cell proliferation and reduced colony formation in a dose dependent manner (Figure 1, Additional file 1). The IC50 values for the ECC-1 and Ishikawa cells were 25 uM and 43 uM after 72 hours of treatment, respectively. We next confirmed that GF was responsible for the activation of the mitochondrial apoptosis pathway, accompanied by an increase in cleaved caspase3 and a decrease in MCL-1 and BCL-2 protein expression (Additional file 2: Figure S1). Cell cycle analysis showed minimal G1 phase arrest in the ECC-1 cells and G2 arrest in Ishikawa cells after 24 hours of treatment (Additional file 3: Figure S2), thus, suggesting that GF induces cell cycle changes by altering different checkpoints in different endometrial cancer cells. After treatment with GF for 24 hours, both cell lines had a reduced ability to adhere to laminin-1 as well as decreased migratory capacity as evaluated by a transwell assay. In addition, E–cadherin increased while Slug proteins decreased after treatment with GF (Additional file 4: Figure S3). GF was also shown to increase reactive oxygen species (ROS) and mitochondrial DNA damage after 24 hours of treatment (Additional file 5: Figure S4), indicating that an increase in ROS production and mitochondrial DNA damage might also be involved in the anti-tumorigenic effects of GF in endometrial cancer cells.

**Figure 2:**
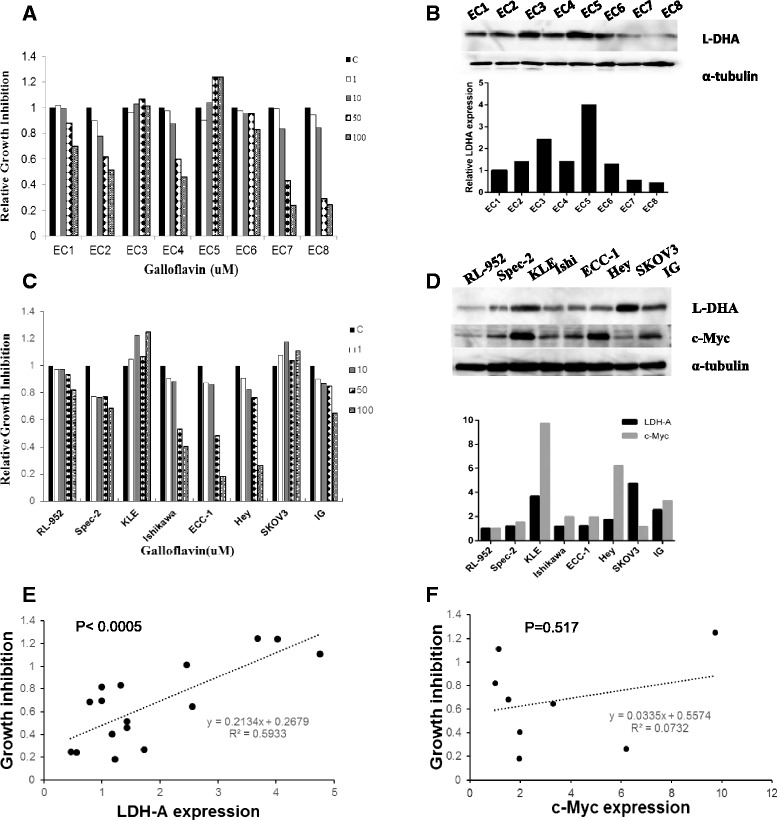
**Galloflavin inhibited cell proliferation in primary cultures of endometrial cancer.** Eight primary cell cultures of endometrial cancer were cultured in 96 well plates and treated with GF as indicated doses for 72 hours. Cell proliferation was assessed by MTT assay **(A)**. LDHA protein expression in the primary cell cultures was detected by Western blotting **(B)**. Eight established endometrial cancer and ovarian cancer cell lines were cultured in 96 well plates, and treated with GF for 72 hours. Cell proliferation was assessed by MTT assay **(C)**. LDHA and c-Myc protein expression in the eight cell lines was detected by Western blotting **(D)**. The relationship of LDHA and c-Myc protein expression and cell response to GF was assessed by Pearson correlation. The results showed a significant correlation between LDHA expression and cell growth inhibition by GF (Pearson r =0.77026; p = 0.0005; n = 16) **(E)**. There was no significant correlation between c-Myc protein expression and cell sensitivity to GF **(F)** (* < 0.05).

Cancer cells maintain a significant level of mitochondrial oxidative phosphorylation (OXPHOS) capacity to rapidly switch from glycolysis to OXPHOS during carcinogenesis and cell energy stress [5,6]. The point of balance between glycolysis and mitochondrial OXPHOS fluctuates depending on changes in the cancer cells microenvironment. Inhibition of LDHA activity by GF resulted in a decreased rate of glucose uptake and ATP production, with subsequent increased pyruvate dehydrogenase (PDH) protein expression and production of pyruvate (Additional file 6: Figure S5). These findings confirm a direct effect of GF on the glucose metabolism by impairing cytosolic glycolysis in the endometrial cancer cells. Since glycolysis and OXPHOS are tightly coupled processes [7], we noted that GF increased glutaminase protein expression, and enhanced Krebs cycle activity, by increasing the production of malate, another Krebs cycle intermediate, after 16 hours of treatment (Additional file 7: Figure S6). GF was effective in inhibition of cell proliferation in 6 of the 8 primary cultures of endometrial cancer with IC50 values ranging from 20-53 uM. A linear regression model showed the level of LDHA protein but not c-Myc was related to the sensitivity to GF in primary cultures and endometrial cancer cell lines (Figure 2). Moreover, we found that GF decreased c-Myc expression in a dose-dependent manner after 24 hours of treatment. Given that c-Myc transcriptionally induces expression and activity of LDHA [8], the inhibition of c-Myc by JQ1 (a c-Myc inhibitor) synergistically increased the inhibitory effects of GF at different concentrations in GF-sensitive and GF-resistant cells (Additional file 7: Figure S6). These results suggest a causal link between GF treatment and down-regulation of c-Myc expression in endometrial cancer cells (Additional file 8: Figure S7). In conclusion, our study indicated that targeted inhibition of LDH by GF has promising anti-tumor activity in endometrial cancer cell lines and primary cultures of endometrial cancer cells. We believe our present study demonstrates that inhibition of LDH by GF decreases cell proliferation, invasion, and glycolytic metabolism while promoting cell stress and apoptosis in endometrial cancer cells. These findings provide a molecular basis for the use of LDH inhibitors in the treatment of endometrial cancer.

## Additional files

Additional file 1:
**Material and Methods.**
Additional file 2: Figure S1. Galloflavin induced apoptosis in ECC-1 and Ishikawa cells. The ECC-1 (A, C) and Ishikawa cells (B, D) were cultured for 24 hours and treated with GF at different concentrations overnight. Apoptosis was examined by an Annexin V assay using Cellometer. The effect of GF on BCL-2, MCL-1, caspase-3 and cleaved caspase-3 was examined by Western blotting in the ECC-1 and Ishikawa cells after exposure of GF for 24 hours at the indicated concentrations (E, F). (* < 0.05). Additional file 3: Figure S2. The effect of Galloflavin on cell cycle progression in the ECC-1 and Ishikawa cells. The ECC-1(A) and Ishikawa (B) were treated with the indicated doses of GF (1–50 uM) for 24 hours. Cell cycle analysis was performed using Cellometer. GF minimally induced cell cycle G1 phase arrest in ECC-1 cells, while Ishikawa exhibited significant G2 phase arrest after GF treatment. Additional file 4: Figure S3. The effect of Galloflavin on adhesion and invasion in theECC-1 and Ishikawa cells. The ECC-1 (A) and Ishikawa (B) cells were cultured for 24 hours and then treated with GF in laminin-1 coated 96 well plates or BME coated 96 transwell plates for 2 hours. GF decreased adhesion and invasion in both cell lines. Western blotting results demonstrated that GF decreased Slug protein expression and increased E-cadherin expression after 24 hours of treatment (C, D). Each experiment was performed three times. (* < 0.05). Additional file 5: Figure S4. Galloflavin induced ROS generation and mitochondrial DNA damage in the ECC-1 and Ishikawa cell lines. The cells were treated with GF at different concentrations for 16 hours. The ROS level was determined using DCFH-DA dye detected on a plate reader in ECC-1 (A) and Ishikawa (B). ECC-1(C) and Ishikawa (D) cells were treated with GF for 24 hours. Mitochondrial DNA damage was analyzed by qPCR assay. (* < 0.05). Additional file 6: Figure S5. Galloflavin inhibited glycolytic metabolism in the endometrial cancer cells. The ECC-1and Ishikawa cells were treated with GF for 2 hours. Glucose uptake was determined using the 2-NBDG assay (A). ATP level, lactate, pyruvate and malate production were determined after treatment of GF for 16 hours (B, C, D. E). Pyruvate dehydrogenase (PDH) and glutaminase (GLS) protein expression were detected by Western blotting. Both PDH and GLS protein expression were increased after 16 hours treatment (* < 0.05). Additional file 7: Figure S6. Inhibition of c-Myc by JQ1 synergistically increased sensitivity of Galloflavin. ECC-1 and Ishikawa cells were cultured for 24 hours and then treated with GF overnight. Western blotting demonstrated that GF inhibited c-Myc protein expression (A). JQ1 synergistically increased the sensitivity of GF after 72 hours treatment in ECC-1 (B), Ishikawa cells (C), SKOV3 (E) and KLE cells (F) (CI < 1). The effect of JQ1 and GF on c-Myc protein expression was assessed by Western blotting in ECC-1, Ishikawa, SKOV3 and KLE cells (D, G). Additional file 8: Figure S7. Postulated pathways by which Galloflavin inhibits LDH activity. GF inhibited LDH activity by either competing for the NADH bind site or inhibiting c-Myc protein expression, or a combination of the two pathways.
