# Supplementary material for: Evaluation of the anti-tumor effects of lactate dehydrogenase inhibitor galloflavin in endometrial cancer cells
Source: J Hematol Oncol. 2015 Jan 29;8:2. doi: 10.1186/s13045-014-0097-x (PMC4316809; doi:10.1186/s13045-014-0097-x)
Supplement: Additional file 1: — Material and Methods. [file 13045_2014_97_MOESM1_ESM.docx]

**Material and Methods**

Cell culture and reagents

The endometrial cancer cell lines, ECC-1, Ishikawa and KLE, were used. The ECC-1 cell line was maintained in RPMI 1640 supplemented with 5% FBS, 300mM L-glutamine, 5 μg/ml bovine insulin, 10,000 U/ ml penicillin and 10,000 μg/ml streptomycin. Ishikawa cells were grown in MEM supplemented 5% FBS, 300 mM L-glutamine, 10,000 U/ml penicillin and 10,000 μg /ml streptomycin. The KLE cell line was maintained in DMED/F12 medium supplemented with 10% FBS. All the cell lines were cultured at 37°C in a humidified atmosphere with 5% CO2.

Galloflavin (GF) was kindly provided by Dr Marinella Roberti (Department of Pharmaceutical Sciences, University of Bologna, Italy). MTT (3-(, 5-dimethylthiazol-2-yl)-2, 5-diphenyltetrazolium bromide) and RNase A were purchased from Sigma (St. Louis, MO). The anti-Pyruvate Dehydrogenase, anti-c-Myc, anti-BCL-2, anti-MCL-1, anti-Caspase-3, anti-Cleved Caspase-3, anti-E-Cadherin, anti-Slug, anti-LDH-A antibodies were purchased from Cell Signaling (Beverly, MA). The anti-Glutaminase antibody was purchased from Abcam (Cambridge, MA). The Annexin V FITC kit was purchased from Biolegend (San Diego, CA). The Enhanced chemiluminescence Western blotting detection reagents were purchased from GE Health care (GE Healthcare Life Sciences, Piscataway, NJ). All other chemicals were purchased from Sigma (St Louis, MO).

MTT proliferation assay

The ECC-1, Ishikawa and KLE cells were plated and grown in 96-well plates at a concentration of 4000 cells/well for 24 hours. These cells were subsequently treated with varying doses of GF or JQ1 for 72 hours. Viable cell densities were determined by metabolic conversion of the dye MTT. MTT (5 mg/ml) was added to the 96-well plates at 5μl/well, followed by an additional hour of incubation. The MTT reaction was terminated through the addition of 100μl of DMSO. The results were read by measuring absorption at 570nm. The effect of GF or JQ1 was calculated as a percentage of control cell growth obtained from DMSO treated cells grown in the same 96-well plates. Each experiment was performed in triplicate and repeated three times to assess for consistency of results.

Colony formation assay

Cells were seeded into 6-well plates at a density of 1000 cells/well in 2 ml of regular media. After 24 hours, the culture media was replaced with fresh media containing 0.1% DMSO as control, or the same media containing 1uM and 50uM GF for 48 hours. The culture media was changed every three days after treatment for two weeks. Colonies were then washed in PBS, fixed with methanol, and stained with crystal violet. Colonies were counted under microscope.

LDH activity assay

Intracellular LDH activity was evaluated using cell lysates and an LDH Activity Assay Kit (Bio Vision, Milpitas, CA) according to the manufacturer’s instructions. Briefly, cells were plated in 6-well cell culture plates (2×105 cells/well) and were treated with varying concentrations GF for 24 hours. Subsequently, the cells were harvested and processed for cell lysate preparation. The protein concentrations in the cell lysates were measured using a BCA Protein Assay Kit (Pierce Biotechnology Inc., Rockford, IL). LDH activity in the cell lysates was determined by monitoring the rate of NADH production from NAD+. After incubation of the samples for 30 min at room temperature, the optical densities at a wavelength of 450nm were analyzed using a micro-plate reader (Tecan, Morrisville, NC). LDH activity was calculated as compared to untreated control. Experiments were performed in triplicate.

Annexin V assay

Annexin V/PI double staining was performed to evaluate the induction of apoptosis. ECC-1 and Ishikawa cells (2 × 105/well) were seeded in 6-well plates. The cells were treated with various concentrations of GF for 24 hours. Then cells were harvested using trypsin, washed with PBS, and suspended in 100μL binding buffer. 1ul of annexin V-FITC (100ug/ml) and 0.5uL of propidium iodide (2mg/ml) were added in the binding buffer in the dark for 15 minutes. Cell apoptosis was detected by Cellometer (Nexelom, Lawrence, MA) and subsequently analyzed by FCS4 express software.

Cell cycle analysis

Cell cycle distribution was determined by staining DNA with PI. Briefly, 6 × 105cells were treated with GF for 48 hours, washed in PBS and then fixed with ice-cold 90% ethanol for 30 minutes. Fixed cells were resuspended in 50ul RNase solution (250 u g/mL) containing 10mM EDTA and incubated at 37°C for 30 minutes. The cells were then stained with PI (10 μ g/mL) in the dark for 15 minutes and detected by Cellometer. The percentage of cells in each cellular phase was analyzed by FCS4 express software.

Reactive oxygen species assay

The molecular probe, 2, 7-Dichlorofluorescein Diacetate (Sigma Aldrich, St. Louis, MO) was used to detect intracellular reactive oxygen species (ROS) production. The ECC-1 and Ishikawa cells were seeded onto a 96-well black culture plate overnight and then the cells were treated with GF at the indicated doses for 16 hours. 10ul DCF-DA (10mM in phenol-free medium) was added into the media for 30 minutes (n=4 per group). The fluorescence intensity was measured at an excitation wavelength of 485 nm and an emission wavelength of 530 nm using a fluorescence micro-plate reader. Data was standardized to the control analysis and then expressed as relative fluorescence units to the controls.

Mitochondrial DNA damage assay

High-molecular-weight DNA was isolated using a kit from QIAGEN (Qiagen Sciences Inc., Germantown, MD) following the manufacturer’s protocol. The concentration of total cellular DNA was determined by using Nanodrop (Tecan, Morrisville, NC). Quantitative PCR (q PCR) assays were performed as previously described with minor modifications [15] The primers for the large fragments of the mtDNA (8.9 kb) were forward 5’-TCA AAG CCT CCT TAT TCG AGC CGA-3’, reverse 5’ – TTT CATCAT GCG GAG ATG TTG GAT GG-3’, and primers for small mtDNA (221bp) fragment are forward 5’ –CCC CAC AAA CCC CAT TAC TAA ACC CA-3’, reverse 5’ – TTT CATCAT GCG GAG ATG TTG GAT GG-3’. A total volume of 50 µl was used in PCRs containing: 15ng of template DNA, 5pmol of each primer, 10× Mix buffer and 2.5 units of recombinant Taq DNA polymerase High Fidelity (Invitrogen Thermo Fisher Scientific Inc., Grand Island, NY). A quantitative control using the half the concentration of control template DNA was included in each set of PCR reactions. Small fragments (211bp) of the mtDNA were also amplified as internal controls. The internal controls were used to normalize the results obtained from the large fragments and to monitor the mitochondrial copy number. The thermal cycling conditions were as follows: 95°C for 3 minutes, followed by 19 cycles of 94°C for 1 minute, 64°C 1 minutes and 68°C for 9 minutes, primer extension at 72 °C for 3 minutes and at the end of these cycles. Every sample was tested in triplicate. qPCR products were quantitated by using the Quant-iT™ dsDNA High Sensitivity Assay Kit (Thermo Fisher Scientific Inc., Grand Island, NY). The average lesion frequency per each fragment was calculated by using the Poisson equation [16].

Adhesion assay

Each well in a 96-well plate was coated with 100ul laminin-1(10ug/ml) and incubated at 37°C for 1 hour. This fluid was then aspirated and 200ul blocking buffer was added to each well for 45-60 min at 37°C. The wells were then washed with PBS and the plate was allowed to chill on ice. To each well, 2.5 x 103 cells were added with PBS and varying concentrations of GF directly. The plate was then allowed to incubate at 37°C for 2 hours. After this period, the medium was aspirated and cells were fixed by directly adding 100ul of 5% glutaraldehyde and incubating for 30 min at room temperature. Adhered cells were then washed with PBS and stained with 100ul of 0.1% crystal violet for 30 minutes. The cells were then washed repeatedly with water, and 100ul of 10% acetic acid was added to each well to solubilize the dye. After 5 minutes of shaking, the absorbance was measured at 570 nm using a micro-plate reader from Tecan (Morrisville, NC).

Invasion assay

Cell invasion assays were performed using 96-well HTS transwells (Corning Life Sciences ,Tewksbury ,MA) coated with 0.5- 1X BME (Trevigen Inc., Gaithersburg, MD). The ECC-1 and Ishikawa cells (50,000/well) were starved for 12 hours and then seeded in the upper chambers of the wells in 50μl FBS-free medium. The lower chambers were filled with 150μl regular medium with GF. The plate was incubated for 24 hours at 37°C to allow invasion into the lower chamber. After washing the upper and lower chambers with PBS once, 100ul Calcein AM solution was added into lower chamber and incubated at 37°C for 30-60 minutes. The lower chamber plate was measured using a plate reader for reading fluorescence at EX/EM 485/520 nm.

Glucose uptake assay

Glucose uptake was measured using 2-NBDG (AAT bioquest, Sunnyvale, CA) after treatment with varying doses GF. 2-NBDG is a fluorescently-labeled deoxyglucose analog that is used primarily to directly monitor glucose uptake by living cells and tissues. The ECC-1 and Ishikawa cells were seeded into 96-well plates at 4000 cells per well overnight and then were treated with 100ul of glucose free culture medium containing 100ug/ml 2-NBDG with varying concentrations of GF (0.1, 10, 50uM) for 20 minutes. The medium was then replaced with 200ul HBSS (Life technologies corporation, Grand Island, NY), and the plate was centrifuged for 5min at 400rpm.This step was then repeated. The fluorescence intensity was measured at an excitation wavelength of 485 nm and an emission wavelength of 530 nm using a plate reader from Tecan (Morrisville, NC).

ATP determination Assay

Cells were seeded in 96-well plates at 4000 cells per well. Varying doses of GF (0–50μM, tested in duplicate) were then added to the culture cells. After 16 hours, ATP levels were measured using an AAT bioquest phosphoWorks tm Luminometric ATP Assay Kit according to the manufacturer’s protocol. In short, 90 ul/well of the ATP Reaction Mix was added into each well, and incubated for 20 minutes at room temperature Luminescence was measured using a micro-plate reader from Tecan (Morrisville, NC). Each experiment was performed in triplicate and repeated twice to assess for consistency of results.

L-lactate Assay

Cells were seeded in 96-well plates at 4000 cells per well overnight. Varying doses of GF (0–50μM) were then added to the cultured cells. After 16 hours of treatment, lactate production in the medium was detected by using the L-Lactate Assay Kit I (Eton Bioscience Inc, San Diego, CA) according to the manufacturer’s protocol. Briefly, 10ul of the culture medium from each well was transferred to a new 96-well plate, 40ul of distilled water was added (bringing the total volume to 50ul). 50ul of lactate assay solution was added to each well and incubated for 30 minutes at 37 ºC without CO2. The intensity of absorbance was measured by using a micro-plate reader from Tecan (Morrisville, NC) at the wavelength of 450 nm.

Pyruvate Assay

Cells were seeded in 96-well plates at 4000 cells per well overnight. Varying doses of GF (0–50μM, tested in duplicate) were then added to the cultured cells. After 16h of treatment, pyruvate production in the medium was detected using a Pyruvate Assay Kit (Assay Biotech, Sunnyvale, CA) according to manufacturer’s protocol. Briefly, 50ul of the culture medium from each sample was transferred to a new 96-well plate. 50ul of working solution was added to each well and then incubated for 30 minutes at 37 ºC .The intensity of fluorescence was measured by using a micro-plate reader from Tecan (Morrisville, NC) at Ex/Em: 540/600.

Malate Assay

Intracellular malate production was evaluated using a Malate Fluorometric Assay Kit (Cayman chemical company, Ann Arbor, MI) according to the manufacturer’s instruction. Briefly, cells were plated in 6-well cell culture plates (2×105cells/well) and were treated with varying concentrations GF for 16 hours. 50ul of sample, 20ul of Malate Fluorometric cofactor, 10ul of developing Enzyme, and 10ul of developer were added into each well. 50ul of Malate Dehydrogenase was added to each well to initiate the reactions. The plate was incubated for 30 minutes at 37ºC. The intensity of fluorescence was measured at Ex/Em: 530/585.

Tissue samples collection and primary cell culture

Eight pathologically diagnosed endometrial cancer samples were collected from UNC Hospital at Chapel Hill. All patients provided written consent, and approval was obtained from the UNC Institutional Review Board (IRB). For the culture of primary endometrial cancer cells, freshly obtained tissues were washed three times with Hank's Buffered Salt Solution (HBSS), and then gently minced by scissors in DMEM/F12 medium containing 10% fetal bovine serum (FBS). Tissues were then digested in 0.2% collagenase IA, 100 U/ml penicillin and anti-anti for 30-60 min hours at 37°C water bath with shaking. After two centrifugations with PBS solution, cells were re-suspended and diluted to 1×105 cells/ml with DMEM/F12 medium. Subsequently, 1×104 cells/well were seeded into 96-well plated and incubated for 24 hours before treated. Cell proliferation was measured with MTT assay 72 hours after treatment.

Western blotting analysis

After JF, JQ1 or combination treatment, cells were washed twice with PBS and then collected and lysed with RIPA-buffer. The protein concentrations in the cell lysates were measured using a BCA Protein Assay Kit (Pierce, Rockford, IL). Protein samples were separated with 12% SDS–PAGE gel and transferred onto the PVDF membranes (GE Healthcare Life Sciences, Piscataway, New Jersey). The membranes were blocked with 5% nonfat dry milk and probed with primary antibodies overnight at 4°C. The membranes then were washed and incubated with a secondary peroxidase conjugated antibody for 1h. Antibody binding was detected using Amersham ECL detection reagents (GE Healthcare Life Sciences, Piscataway, New Jersey) via Alpha Innotech Imaging System (Protein Simple, Santa Clara, CA). All blots were stripped and reprobed with polyclonal anti-β-actin antibody or anti-α-tubulin antibody to ascertain equal loading of proteins. Each experiment was repeated three times to assess for consistency of results.

Statistical analysis

All statistical analysis in this study was carried out using Graphpad prism (Graphpad, SanDiego, CA). A p value of 0.05 or less was considered as statistically significant. Statistical analysis on synergy was used to evaluate the effects of combined drug treatments. The results from the MTT assays of each cell line after treatment with GF alone, JQ1 alone and the combination of these two agents were analyzed by the CalcuSyn for Windows computer program (Biosoft, Cambridge, UK) to determine the presence of synergy between JQ1 and GF. CI < 1 indicates synergistic activity; whereas a CI value = 1 and a CI > 1 indicate additive and antagonistic effects, respectively.
