# Supplementary figures and images for: Evaluation of the anti-tumor effects of lactate dehydrogenase inhibitor galloflavin in endometrial cancer cells
Source: J Hematol Oncol. 2015 Jan 29;8:2. doi: 10.1186/s13045-014-0097-x (PMC4316809; doi:10.1186/s13045-014-0097-x)

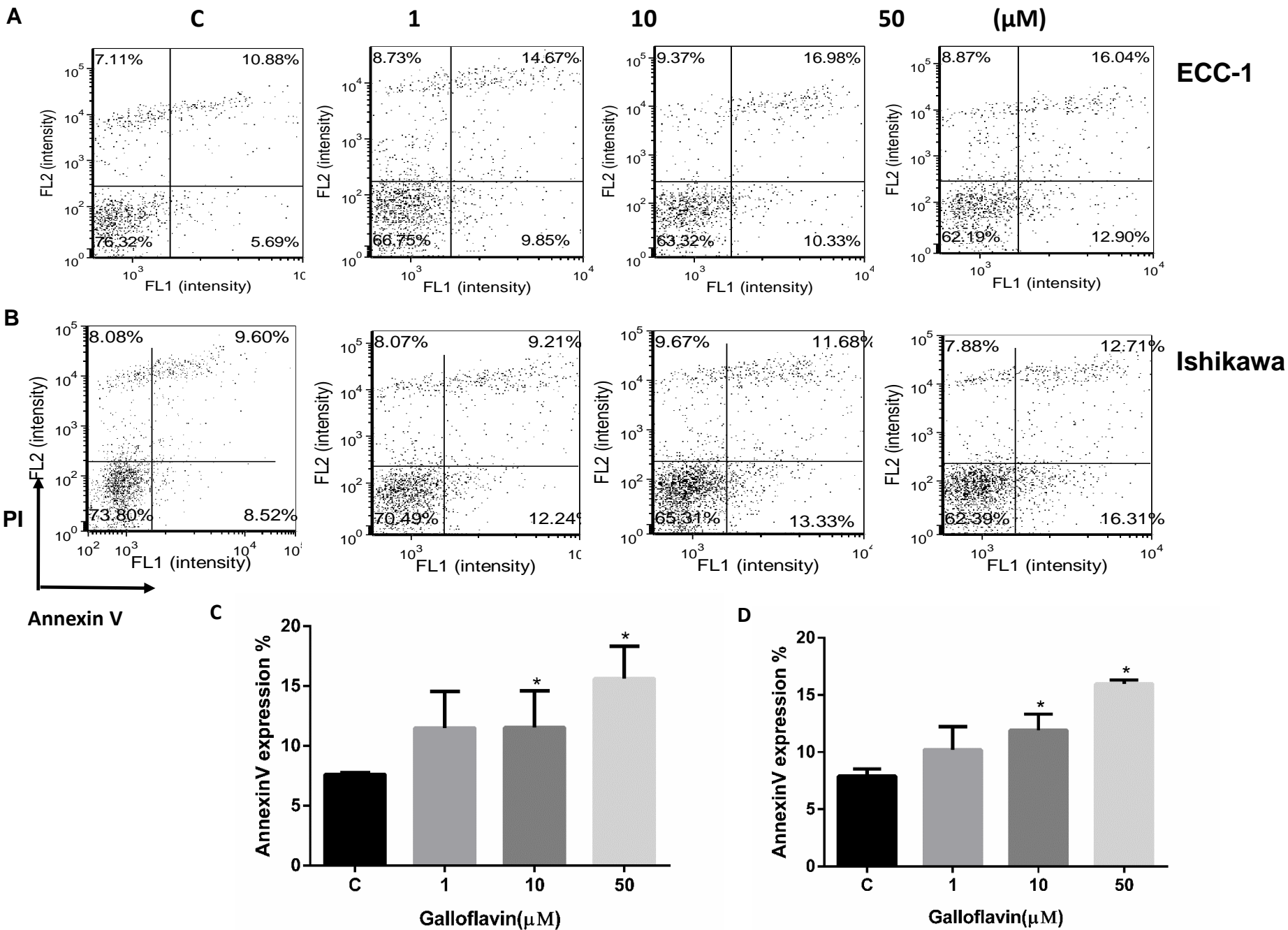

**E**

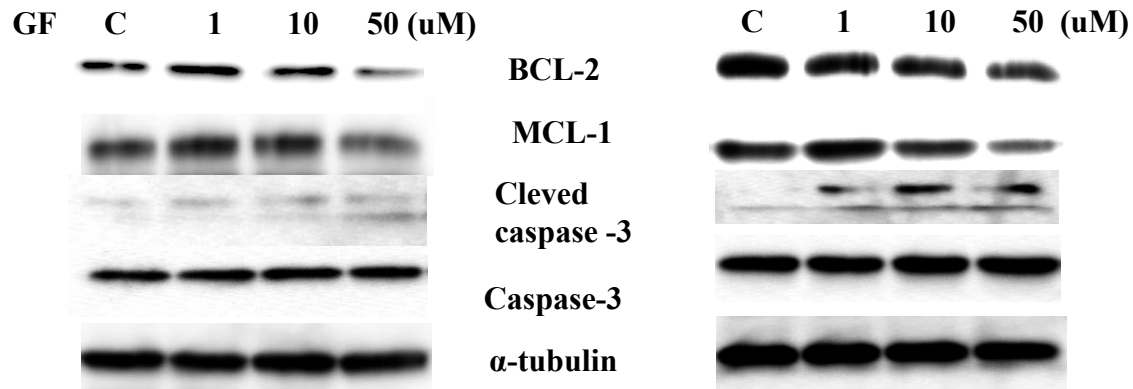

**ECC-1**

**Ishikawa**

**F**

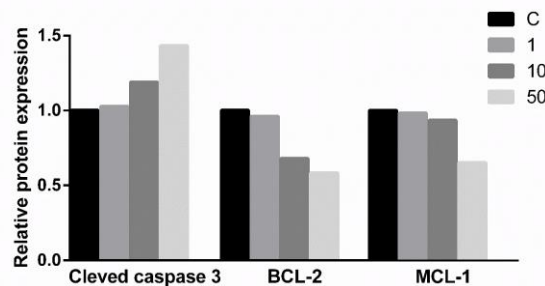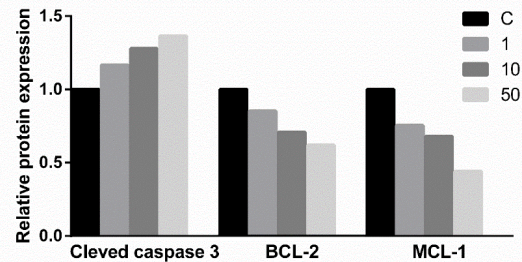

Supplement: Additional file 2: Figure S1. — Galloflavin induced apoptosis in ECC-1 and Ishikawa cells. The ECC-1 (A, C) and Ishikawa cells (B, D) were cultured for 24 hours and treated with GF at different concentrations overnight. Apoptosis was examined by an Annexin V assay using Cellometer. The effect of GF on BCL-2, MCL-1, caspase-3 and cleaved caspase-3 was examined by Western blotting in the ECC-1 and Ishikawa cells after exposure of GF for 24 hours at the indicated concentrations (E, F). (* < 0.05). [file 13045_2014_97_MOESM2_ESM.pdf]

A

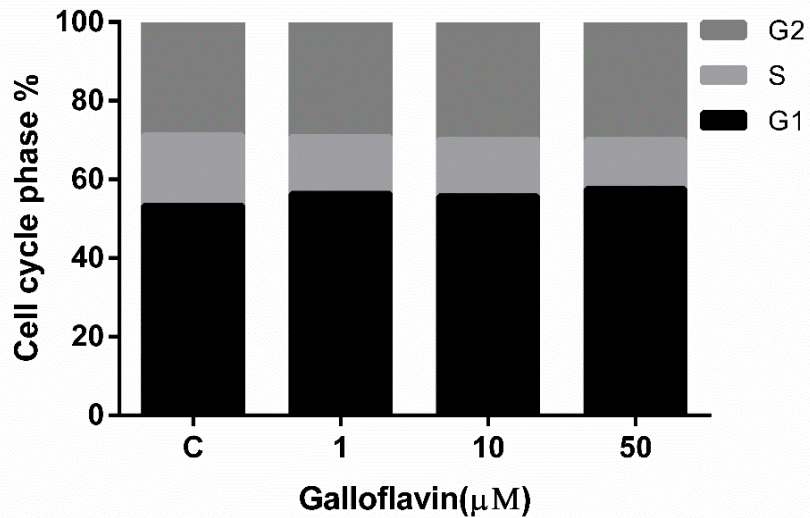

B

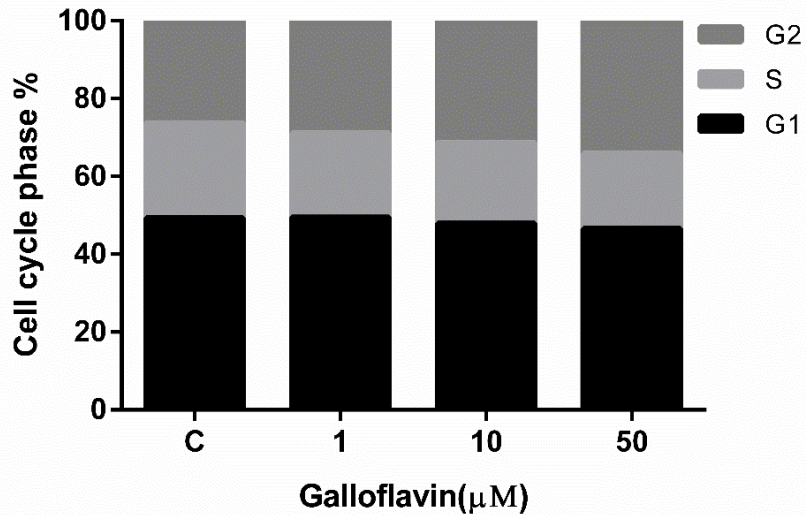

Supplement: Additional file 3: Figure S2. — The effect of Galloflavin on cell cycle progression in the ECC-1 and Ishikawa cells. The ECC-1(A) and Ishikawa (B) were treated with the indicated doses of GF (1–50 uM) for 24 hours. Cell cycle analysis was performed using Cellometer. GF minimally induced cell cycle G1 phase arrest in ECC-1 cells, while Ishikawa exhibited significant G2 phase arrest after GF treatment. [file 13045_2014_97_MOESM3_ESM.pdf]

A

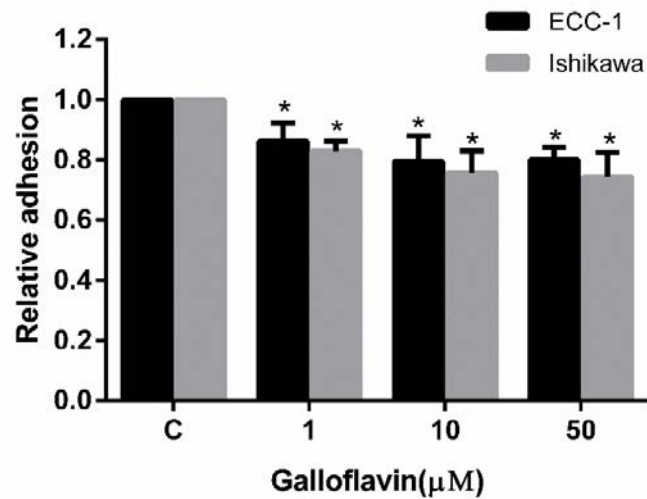

B

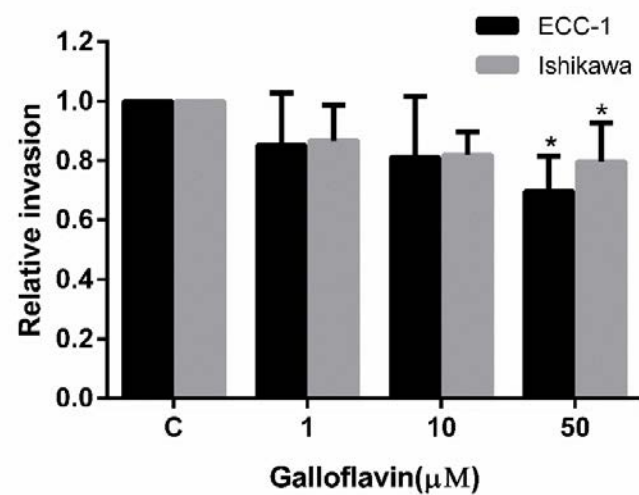

C

GF C 1 10 50 ( $\mu$ M)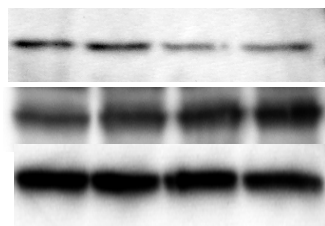

Slug  
E-Cadherin  
 $\alpha$ -tubulin

D

ECC-1

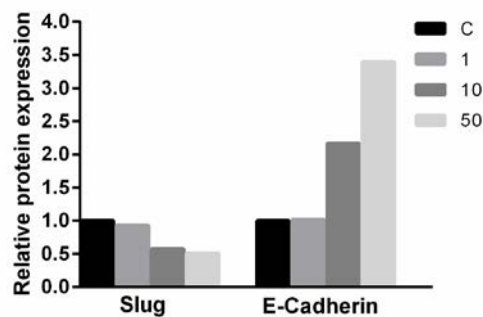

Ishikawa

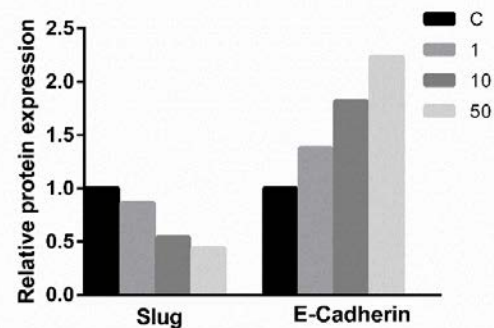

Supplement: Additional file 4: Figure S3. — The effect of Galloflavin on adhesion and invasion in theECC-1 and Ishikawa cells. The ECC-1 (A) and Ishikawa (B) cells were cultured for 24 hours and then treated with GF in laminin-1 coated 96 well plates or BME coated 96 transwell plates for 2 hours. GF decreased adhesion and invasion in both cell lines. Western blotting results demonstrated that GF decreased Slug protein expression and increased E-cadherin expression after 24 hours of treatment (C, D). Each experiment was performed three times. (* < 0.05). [file 13045_2014_97_MOESM4_ESM.pdf]

**A**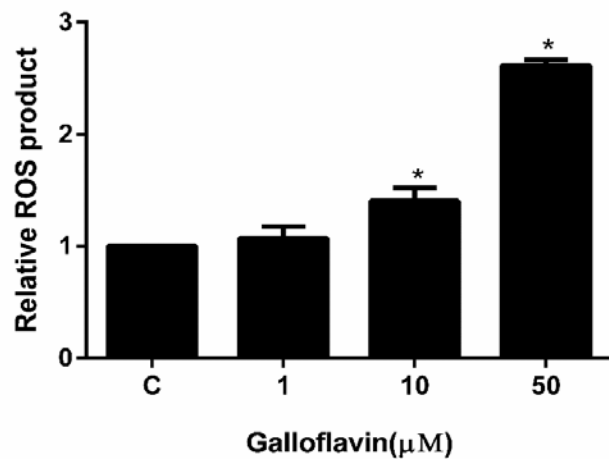**B**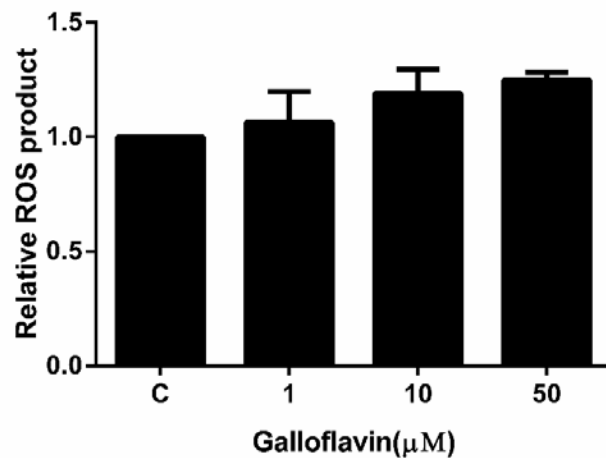**C**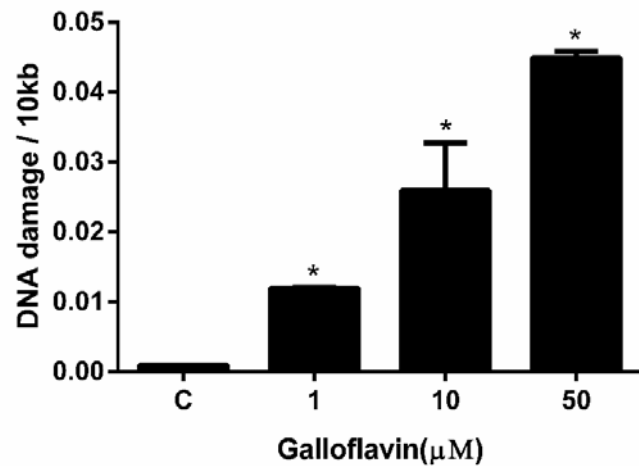**D**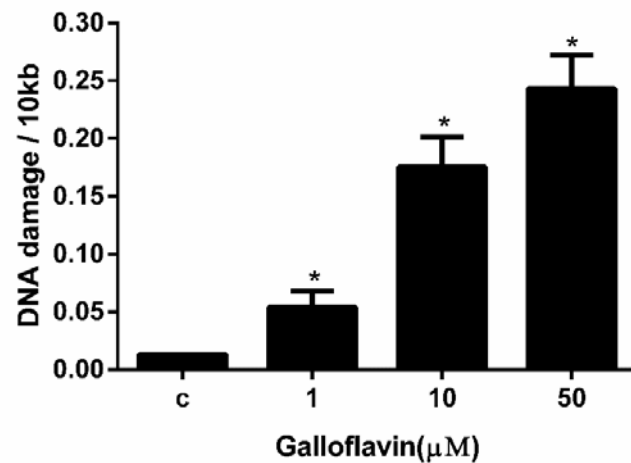

Supplement: Additional file 5: Figure S4. — Galloflavin induced ROS generation and mitochondrial DNA damage in the ECC-1 and Ishikawa cell lines. The cells were treated with GF at different concentrations for 16 hours. The ROS level was determined using DCFH-DA dye detected on a plate reader in ECC-1 (A) and Ishikawa (B). ECC-1(C) and Ishikawa (D) cells were treated with GF for 24 hours. Mitochondrial DNA damage was analyzed by qPCR assay. (* < 0.05). [file 13045_2014_97_MOESM5_ESM.pdf]

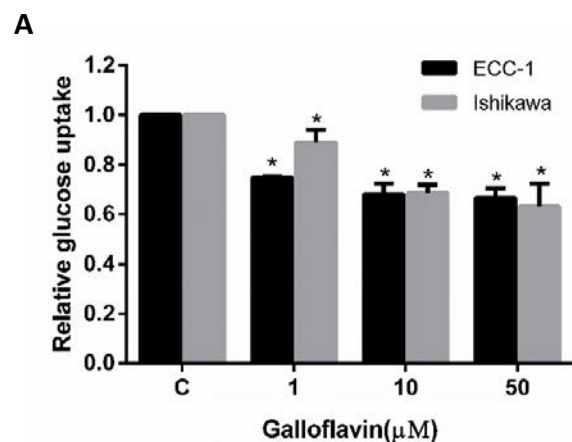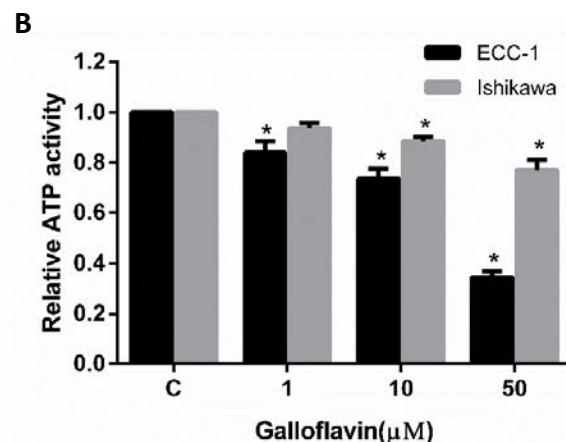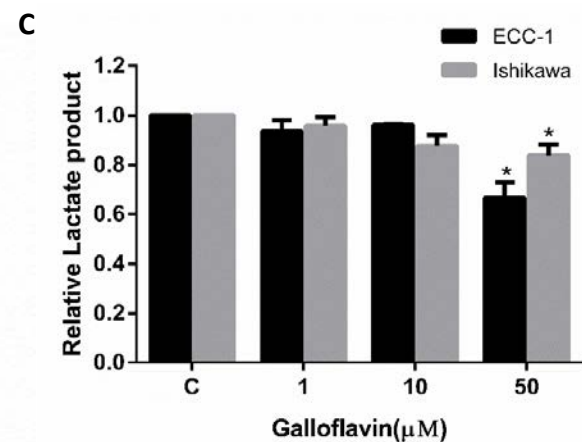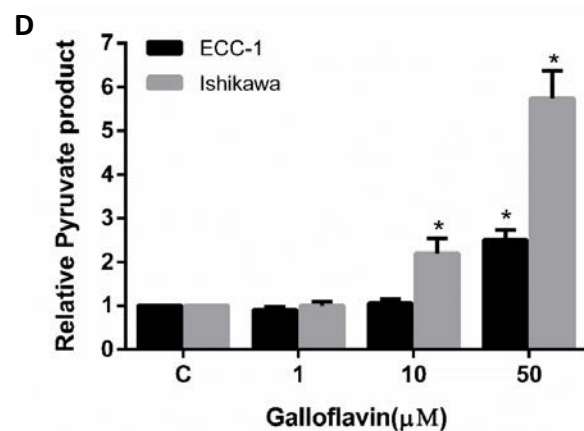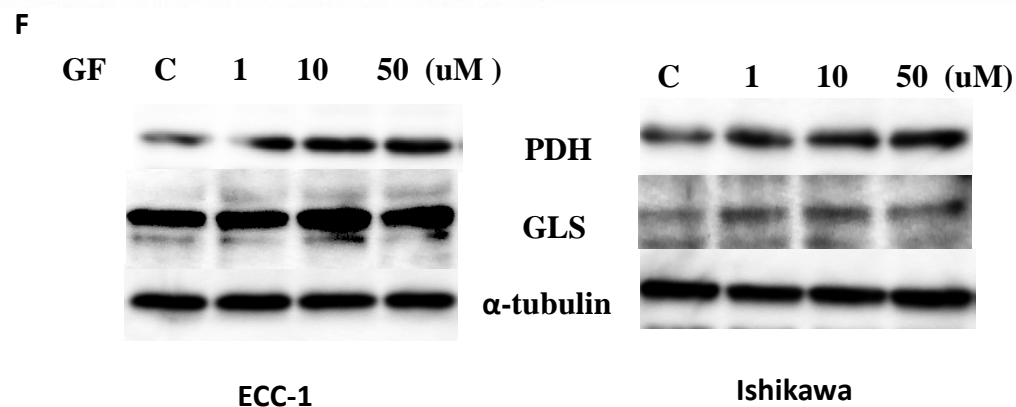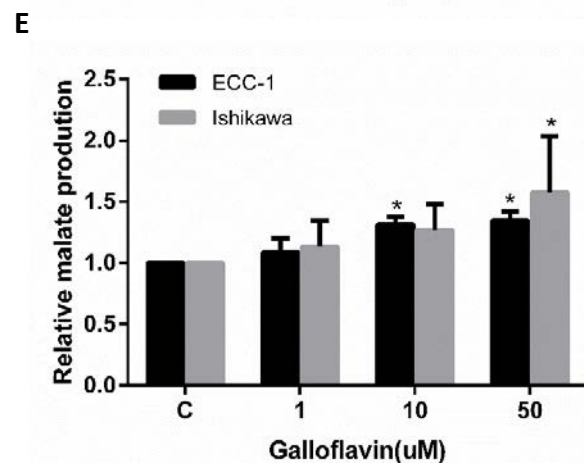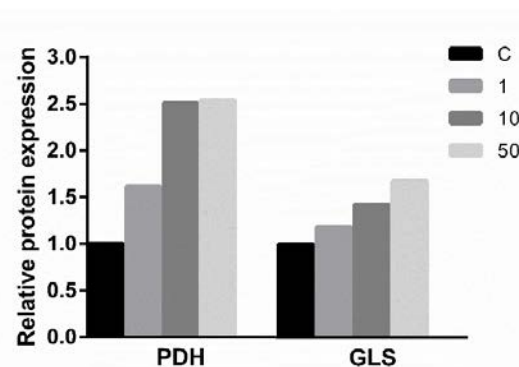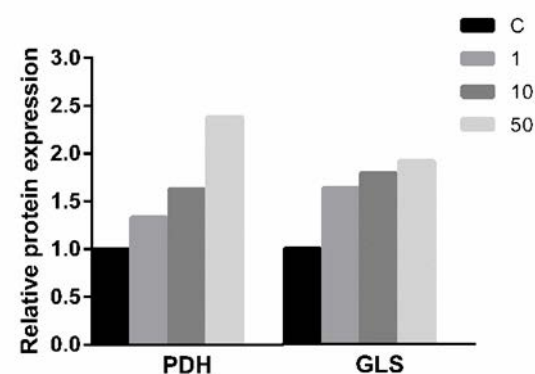

Supplement: Additional file 6: Figure S5. — Galloflavin inhibited glycolytic metabolism in the endometrial cancer cells. The ECC-1and Ishikawa cells were treated with GF for 2 hours. Glucose uptake was determined using the 2-NBDG assay (A). ATP level, lactate, pyruvate and malate production were determined after treatment of GF for 16 hours (B, C, D. E). Pyruvate dehydrogenase (PDH) and glutaminase (GLS) protein expression were detected by Western blotting. Both PDH and GLS protein expression were increased after 16 hours treatment (* < 0.05). [file 13045_2014_97_MOESM6_ESM.pdf]

**A**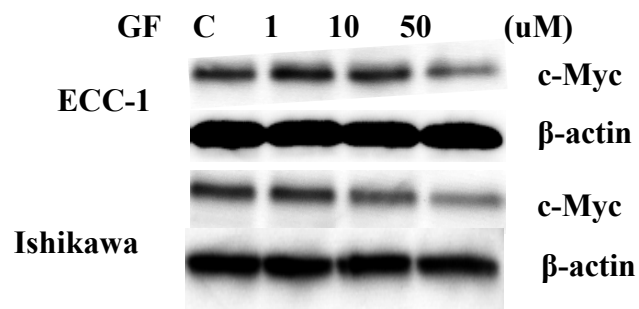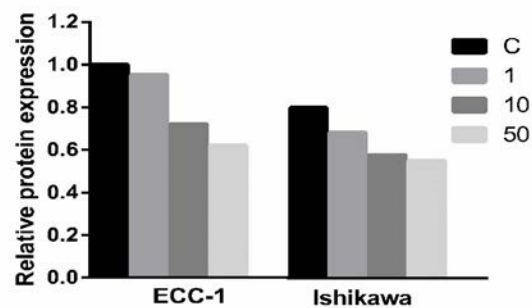**B**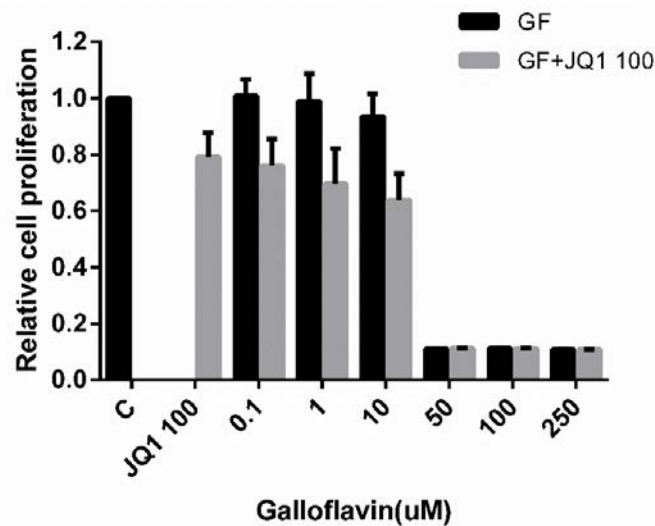**C**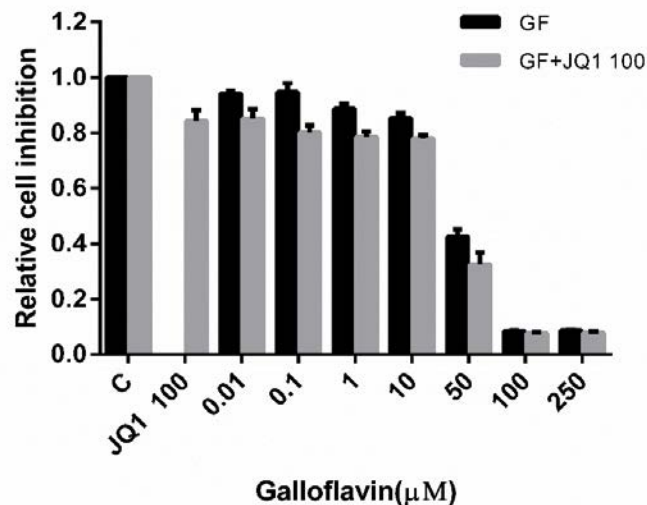**D**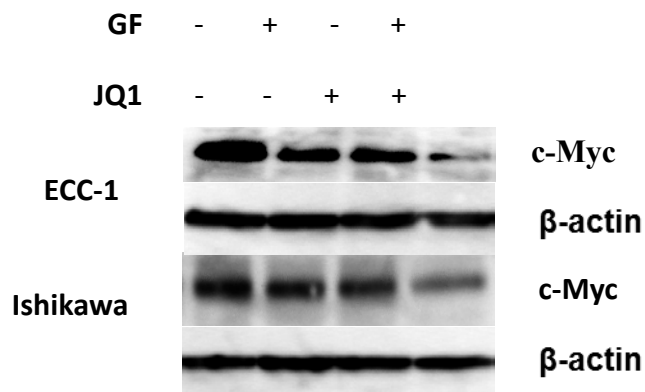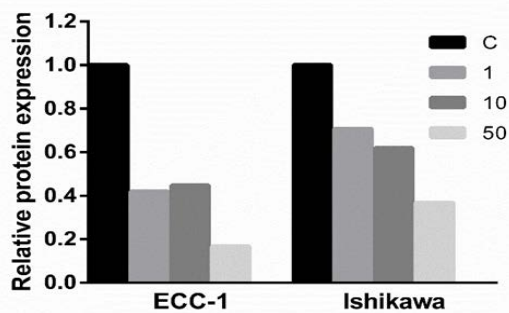

E

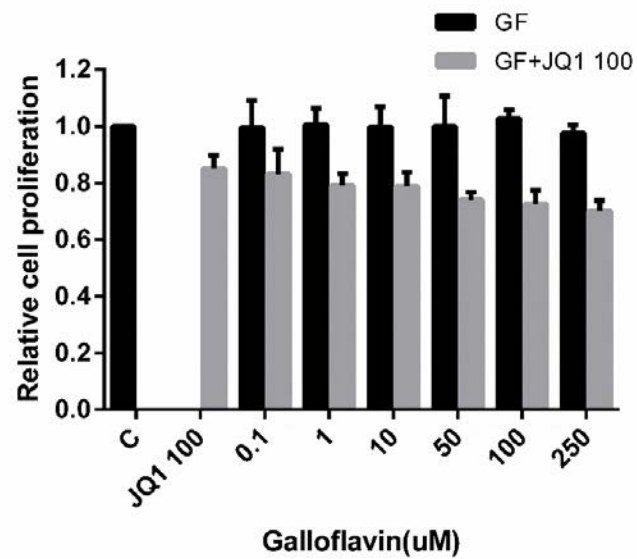

F

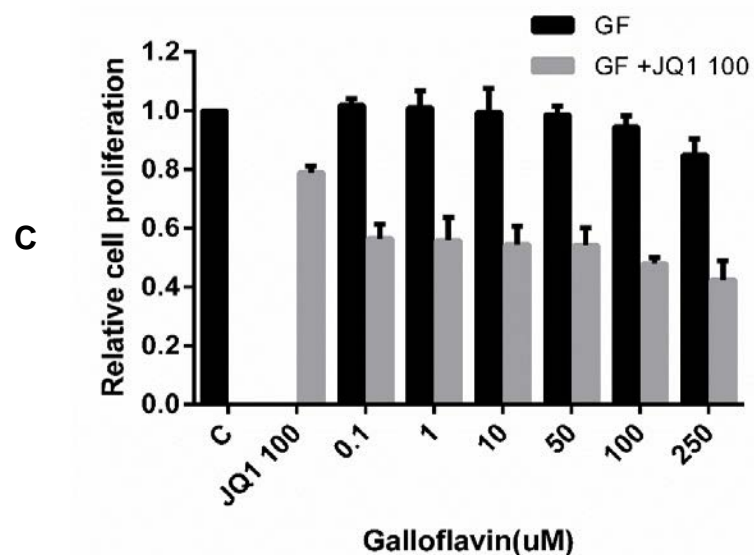

C

G

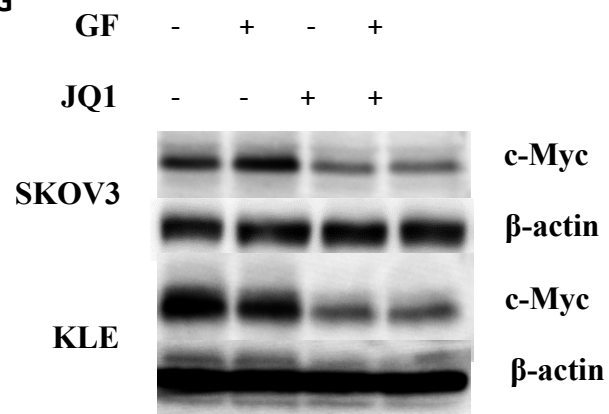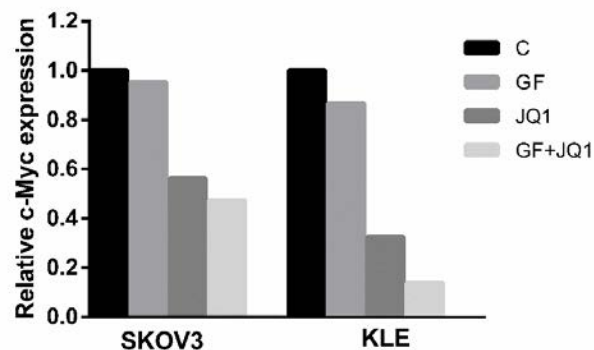

Supplement: Additional file 7: Figure S6. — Inhibition of c-Myc by JQ1 synergistically increased sensitivity of Galloflavin. ECC-1 and Ishikawa cells were cultured for 24 hours and then treated with GF overnight. Western blotting demonstrated that GF inhibited c-Myc protein expression (A). JQ1 synergistically increased the sensitivity of GF after 72 hours treatment in ECC-1 (B), Ishikawa cells (C), SKOV3 (E) and KLE cells (F) (CI < 1). The effect of JQ1 and GF on c-Myc protein expression was assessed by Western blotting in ECC-1, Ishikawa, SKOV3 and KLE cells (D, G). [file 13045_2014_97_MOESM7_ESM.pdf]

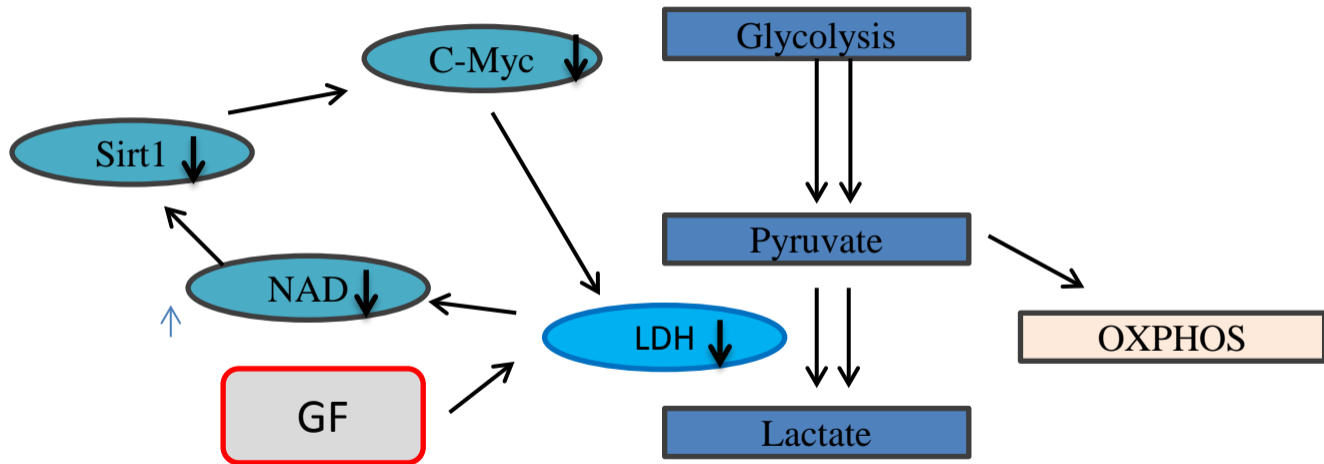

Supplement: Additional file 8: Figure S7. — Postulated pathways by which Galloflavin inhibits LDH activity. GF inhibited LDH activity by either competing for the NADH bind site or inhibiting c-Myc protein expression, or a combination of the two pathways. [file 13045_2014_97_MOESM8_ESM.pdf]
